# Classification of Parkinson's disease stages with a two-stage deep neural network

**DOI:** 10.3389/fnagi.2023.1152917

**Published:** 2023-06-02

**Authors:** José Francisco Pedrero-Sánchez, Juan Manuel Belda-Lois, Pilar Serra-Añó, Sara Mollà-Casanova, Juan López-Pascual

**Affiliations:** ^1^Instituto de Biomecánica (IBV), Universitat Politècnica de València, Valencia, Spain; ^2^Department of Mechanical and Materials Engineering (DIMM), Universitat Politècnica de València, Valencia, Spain; ^3^UBIC, Department of Physiotherapy, Faculty of Physiotherapy, Universitat de València, Valencia, Spain

**Keywords:** Parkinson's disease, classification severity, neural network, smartphone, functional assessment

## Abstract

**Introduction:**

Parkinson's disease is one of the most prevalent neurodegenerative diseases. In the most advanced stages, PD produces motor dysfunction that impairs basic activities of daily living such as balance, gait, sitting, or standing. Early identification allows healthcare personnel to intervene more effectively in rehabilitation. Understanding the altered aspects and impact on the progression of the disease is important for improving the quality of life. This study proposes a two-stage neural network model for the classifying the initial stages of PD using data recorded with smartphone sensors during a modified Timed Up & Go test.

**Methods:**

The proposed model consists on two stages: in the first stage, a semantic segmentation of the raw sensor signals classifies the activities included in the test and obtains biomechanical variables that are considered clinically relevant parameters for functional assessment. The second stage is a neural network with three input branches: one with the biomechanical variables, one with the spectrogram image of the sensor signals, and the third with the raw sensor signals.

**Results:**

This stage employs convolutional layers and long short-term memory. The results show a mean accuracy of 99.64% for the stratified k-fold training/validation process and 100% success rate of participants in the test phase.

**Discussion:**

The proposed model is capable of identifying the three initial stages of Parkinson's disease using a 2-min functional test. The test easy instrumentation requirements and short duration make it feasible for use feasible in the clinical context.

## 1. Introduction

Parkinson's disease (PD) is a prevalent progressive neurodegenerative disease (Ascherio and Schwarzschild, 2016; Simon et al., 2020). In the advanced stages, PD can cause motor dysfunction that alters the performance of basic activities of daily living (ADLs). Early identification of PD through clinical evaluation and functional tests allows the healthcare personnel to intervene properly in rehabilitation plans (Ascherio and Schwarzschild, 2016). Understanding the specific functional alterations in ADL, such as balance, gait, sitting, or standing, can help clinicians develop individualized rehabilitation plans and improve the quality of life of PD patients (Ascherio and Schwarzschild, 2016).

In the recent years there has been a trend toward sensorizing and applying data processing techniques to clinical functional tests. Portable sensors such as instrumented insoles, accelerometers, or inertial sensors (Ponciano et al., 2020) have been used to obtain clinically relevant parameters for studying the functional alterations of PD patients (Serra-Añó et al., 2020; Mollà-Casanova et al., 2022). The use of instrumented functional tests have also resulted in the generation of significant amounts of data (Weiss et al., 2011; Channa et al., 2020; Fuentes-Abolafio et al., 2020), opening up the possibility of applying advanced data analysis techniques such as machine learning and deep learning (Rehman et al., 2019; Butt et al., 2020; Xia et al., 2020; Mirelman et al., 2021).

In PD, clinically relevant parameters obtained from functional tests have been used to generate mathematical models that establish disease severity classifications (Bhidayasiri and Tarsy, 2012), determine functional status categories (Wrisley and Kumar, 2010), or identify risk levels (Sun and Sosnoff, 2018; Friedrich et al., 2021). Many studies have focused on analysing signals in the space-time domain, calculating biomechanical variables such as the trajectory of the center of pressures or time distribution during gait phases (Tong et al., 2021). Various classification techniques, including support vector machine (SVM), random forest (RF), decision trees (DT), or k-nearest neighbors (KNN; Trabassi et al., 2022), have been used to classify the severity of Parkinson's disease with an accuracy around 80 and 90%.

Although discrete variables-based methods have shown good results, they have a significant disadvantage of requiring prior feature selection and signal parametrization. This process is time-consuming and may lead to the loss of valuable information. These drawbacks may be overcome using the sensor raw data as the input to an artificial neural network (ANN), letting the ANN itself to identify the relevant information and extract the features to build the model. This approach has already shown very good results in the classification of PD severity, with an accuracy between 95 and 98%, using convolutional neural networks (CNN; El Maachi et al., 2020), long short-term memory (LSTM; Zhao et al., 2018a; Butt et al., 2020), or a combination of both (Zhao et al., 2018b; Xia et al., 2020).

Some authors have explored the analysis in the frequency domain instead of the time domain (Kim et al., 2018). The processed the spectrogram image of inertial sensors recordings using CNN, hypothesizing that the frequency components of involuntary movements could aid in identifying the level of severity of the disease. Although the accuracy rate in classifying PD stages was lower with this frequency analysis approach (83–85%) compared to the time domain approach, it may provide complementary information valuable for clinical evaluation of PD.

Considering the aforementioned findings, we hypothesize that a mixed input model comprising all three types of data (biomechanical variables, time domain, and frequency domain) would be capable of extracting all the relevant clinical features, outperforming the accuracy of simpler models.

The main objective of this study is to assess the accuracy of a mixed input model for classifying the early stages of PD using an instrumented functional assessment test. To achieve this, we developed a two-stage model that employs biomechanical variables, sensor raw data, and frequency analysis as inputs. We compared the performance of the proposed model was with that of simpler models that only utilized a subset of the inputs (raw signals only, frequency analysis only, and biomechanical variables only). As a secondary objective, we tested the accuracy of a CNN in automating the process of signal semantic segmentation and biomechanical variables calculation from the sensor raw data.

## 2. Materials and methods

### 2.1. Participants

Eighty-seven participants with PD distributed according to the Hoehn and Yahr (HY) scale (21 stage I, 30 stage II, and 36 stage III) agreed to participate in this cross-sectional study. Inclusion criteria for participation in the study has been as follows: (i) PD diagnosed by a neurologist [HY I, II, and III] (Hoehn and Yahr, 1967), (ii) have optimized and stable medical therapy at least one month before enrolment; (iii) have good cognitive status, defined as a score higher than 23 on the Mini-Mental State Exam (Folstein et al., 1975), (iv) ability to perform a modified Timed up & go (TUG) independently.

Exclusion criteria has been: (i) medical contraindications to physical activity, (ii) neurological or orthopedic injuries limiting independent walking and sitting or standing up from a chair, (iii) deafness or hearing problems, (iv) vestibular impairment, (v) blindness or a visual impairment, (vi) mental illness, (vii) any surgical procedure within the past 6 months before enrolment; (viii) people with IV and V stages of PD.

Participants were prospectively classified using the HY scale by their referring neurologist. Then, a physiotherapist conducted the functional assessment proposed, and scored the participant again on the HY scale. Stages IV and V were excluded from the study due to the implied severe disability that made it difficult to perform the test independently without the use of assistive products (Giladi et al., 2001; Goetz et al., 2004; Lescano et al., 2016).

All procedures were conducted in agreement with the World Medical Association Declaration of Helsinki principles. Ethical approval for the study was granted by the Ethics Committee of Universitat de València (H1517239006520), and all volunteers that participated in the study provided written informed consent.

### 2.2. Functional assessment

**Figure 1:**
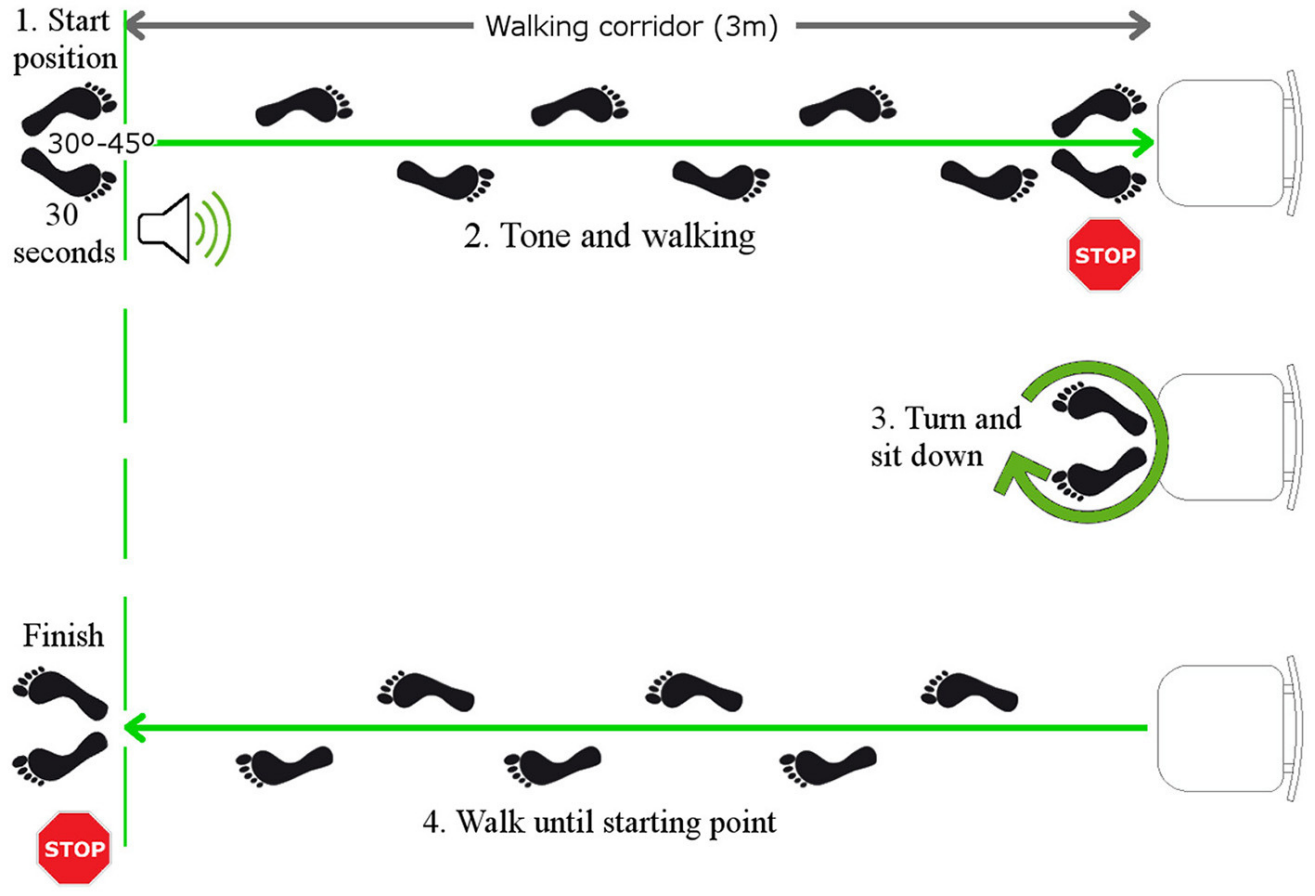
Functional assessment test execution sequence. 1. Balance standing upright for 30 s until the sound stimulus sounds; 2. Walk in a straight line toward the chair located 3 m away; 3. Turn around and sit in the chair; 4. Walk 3 m to the starting area and end the recording of the functional test.

The functional assessment test is based on a modification of the TUG test already used and validated in this type of population (Serra-Añó et al., 2020; Mollà-Casanova et al., 2022). The modification to the TUG consists on: the inclusion of a pre-balance phase, the assessment of the reaction time to an external sound stimulus (Serra-Añó et al., 2019). The assessment of sitting-up and standing-up from a chair. The test consists of the following four phases (Figure 1):

- **Phase 1:** bipodal balance for 30 s with arms alongside the body.
- **Phase 2:** walking in a straight line toward a chair 3 m away when the external sound stimulus is produced.
- **Phase 3:** turn around and sit on the chair, get up from the chair.
- **Phase 4:** walk 3 m back to the starting area.

The participants were asked to perform the protocol as quickly as possible while staying within their safety margins to avoid any possible harm. The test was conducted using an inertial sensor embedded in an Android smartphone (High Performance 6-Axis MEMS MotionTrackingTM composed of 3-axis gyroscope; 3-axis accelerometer at 100 Hz) attached to the back of the waist (L4-L5 vertebrae) with a strap. Throughout the study, the sensor signals were recorded using the Fallskip^Ⓡ^ system app. FallSkip^Ⓡ^ is a commercial system developed by the IBV (Instituto de Biomecánica de Valencia). This system was solely used in our study for recording the measurements and controlling the testing times. No calculations or analysis were performed by the FallSkip^Ⓡ^ application. Instead, all the calculations and analysis were performed offline on dedicated scripts for the analysis of the data.

### 2.3. Model data flow

**Figure 2:**
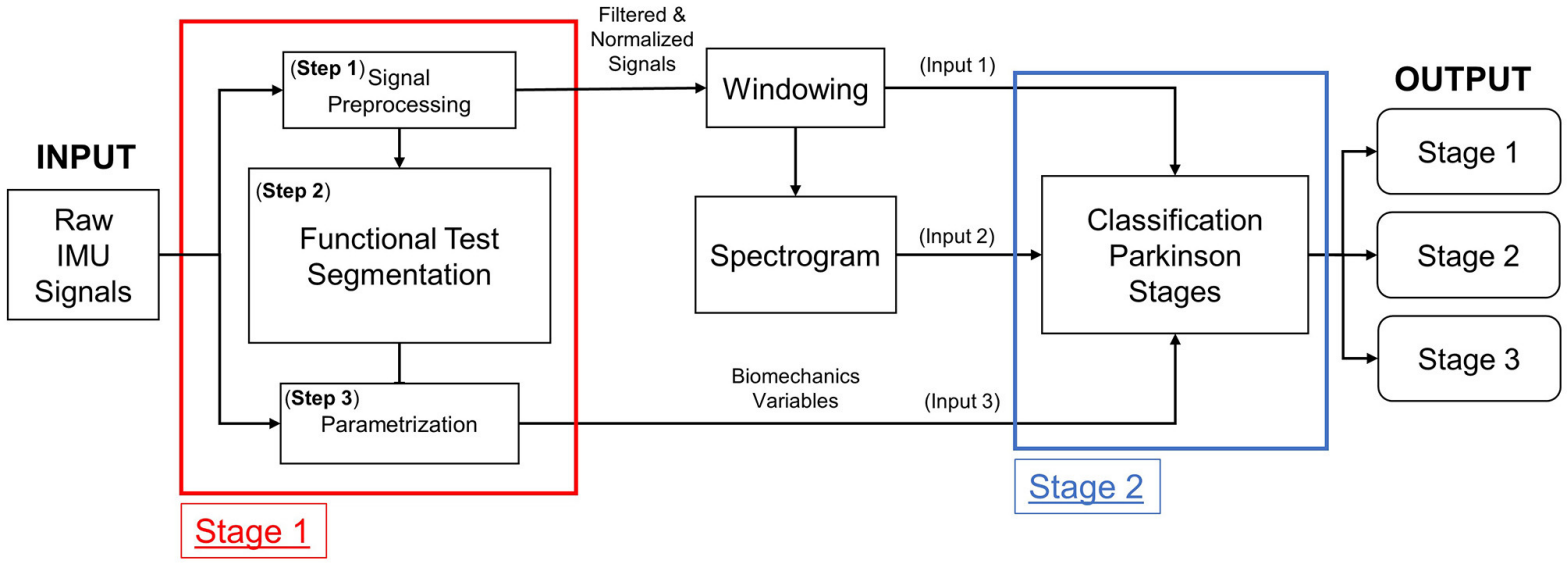
Structure of the two-stage Parkinson classifier model.

A two-stage model has been designed (Figure 2). The raw sensor signals are the input of **Stage 1**, where are filtered and normalized in a first step (**Step 1**) before running the automatic segmentation of the test phases at step 2 (**Step 2**) which delivers the start and end times of each phase. Finally, the biomechanical variables are computed in step 3 (**Step 3**; Mollà-Casanova et al., 2022). The classification model based on neural networks of mixed input data is implemented in **Stage 2**. Each input branch of the model characterizes one aspect of the input signal: (**Input 1**) time-domain analysis, (**Input 2**) frequency-domain analysis (from the spectrogram), and (**Input 3**) biomechanical variables selected from literature (Serra-Añó et al., 2020; Mollà-Casanova et al., 2022). All this information is concatenated into a model (**Stage 2**) that classifies into the first three Parkinson's stages.

In the following sections, each of the processes that comprise the proposed two-stage model are described. All data processing were written in Python (v3.X).

### 2.4. Stage 1

#### 2.4.1. Step 1—Signal preprocessing

Signal processing was carried out following the methodology proposed in Pedrero-Sánchez et al. (2022) which builds on the work of Zijlstra (2004) and Nishiguchi et al. (2012) for analyzing the data from inertial sensors. First, a linear interpolation was applied to standardize the sampling frequency of all signals to 100 Hz. Next, a 4th-order zero-lag Butterworth low-pass filter with a cutoff frequency of 20 Hz was applied. Then, we used the MinMaxScaler preprocessing function from the SciKitLearn library (Pedregosa et al., 2011) to normalize each signal between −1 and 1.

Before segmenting the functional test with the model, we employed a sliding window process because the segmentation model uses convolutional layers that require input data of uniform shape. Specifically, we applied a 64-sample moving window to the six sensor signals (three axes of accelerometer and three axes of gyroscope) to produce a matrix of shape 64 timestamps by six signals. The sliding window was then shifted through the entire signal, overlapping by 63 samples.

#### 2.4.2. Step 2—Functional test segmentation

To automatically segment the different phases of the functional test, a 1D Unet model was set up. This model is necessary to calculate the features of the sensor signals before passing them as input to the classification model. Typically, semantic segmentation RNN models have an Encoder-Decoder structure, where the input and output have the same shape. A forward feedback is performed between the layers forming a Unet structure (Ronneberger et al., 2015). The segmentation model proposed by Ronneberger was originally designed to segment images, but for this study, the internal structure of each encoding and decoding block has been modified to work with 1D vectors.

**Figure 3:**
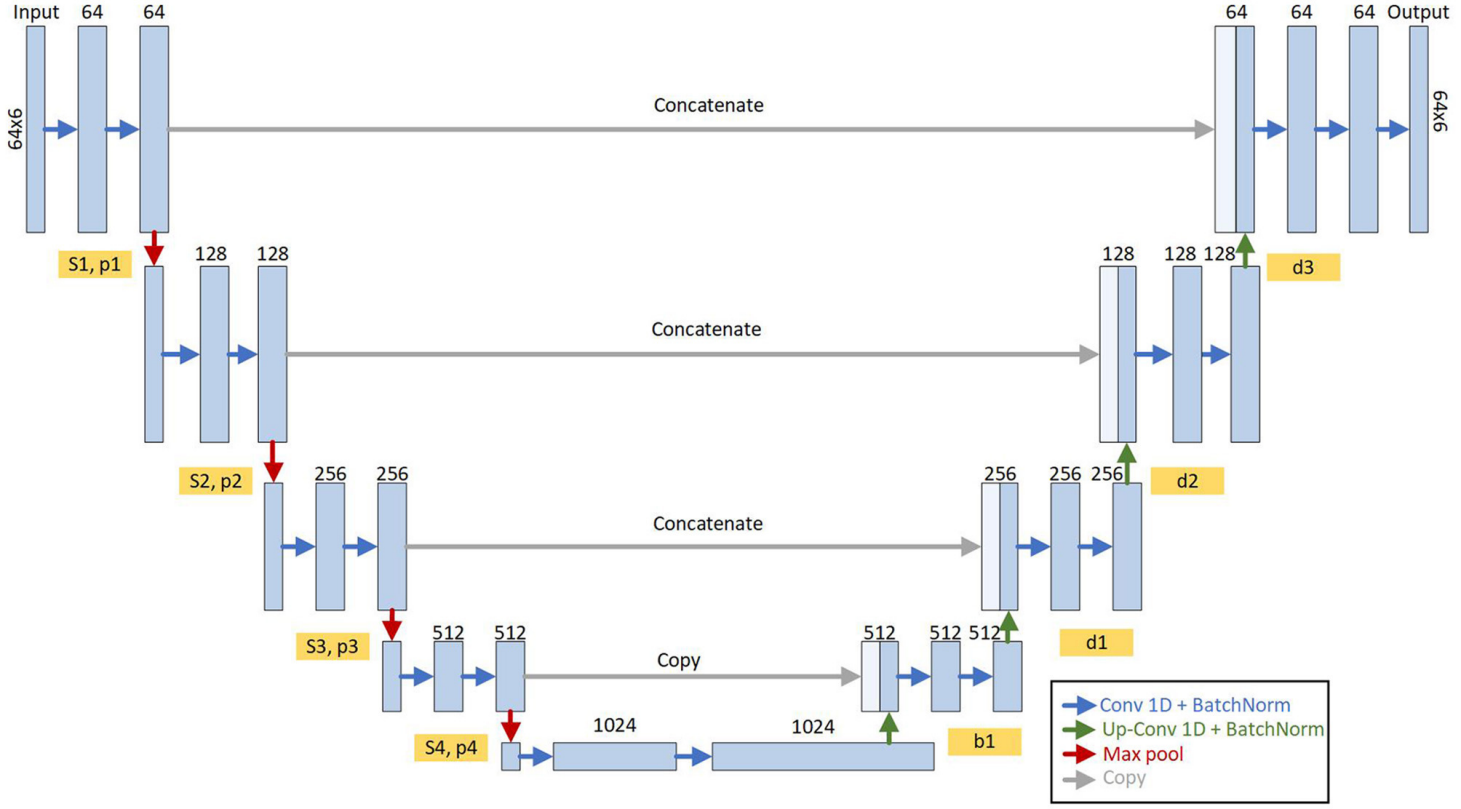
Structure of the Unet model for semantic segmentation of functional assessment. It is composed of four encoder blocks and four decoder blocks interconnected with a bridge in the central part where all the characteristics of the input signals are encoded. Each encoder/decoder block is composed of a series of 1D convolutional layers and a normalization (blue arrows). The outputs of these blocks (Sn and Pn) are interconnected with the next encoder block (red arrows) and with the analog decoder (gray arrows). The output of the model is the probability of each timestamp (64 input timestamps) of the activity of the functional test.

The structure of the model is depicted in Figure 3, where the input consists of the sliding windows from Step 1 (Section 2.4.1). The output has a shape 64 samples by 6 possible categories, corresponding to each of the possible phases of the test: balance, walking, turning and sitting, sitting, getting up, and a noise category.

Given that the model outputs an activity type for each sample in the window, we opted to identify the activity within the window by choosing the activity with the highest frequency as the identified activity. Then, once we identified all the activities in each sample of the complete functional test, we proceeded to detect the start and end instants of each phase of the test where the changes in activity occurred.

The model was developed from scratch, with the Adam optimizer, a learning rate of 0.001, and “categorical crossentropy” as the loss function. The Adam optimizer (Bock and Weiss, 2019) is the most widely used variation of gradient descent algorithms.

#### 2.4.3. Step 3—Signal features

The input features calculated for the model (Step 3) have been previously validated in studies such as Ribeiro et al. (2003), Zijlstra (2004), Esser et al. (2009), and Nishiguchi et al. (2012). The features included are:

- **Phase 1, balance:** range of the Medial-Lateral Displacement (MLDisp) of the Center Of Mass (COM); range of Anterior-Posterior Displacement (APDisp) of the COM; and Swept Area (DispA).
- **Phase 2 and 4, gait:** range of the Vertical displacement (Vrange) of the COM; range of the Medial-Lateral displacement (MLRange) of the COM.
- **Phase 3, turn-to-sit-to-stand:** Turn-to-sit power (PTurnSit); Sit-to-stand power (PStand) (Lindemann et al., 2003); range of jerk to sit (JerkSit); range of jerk to stand (JerkStand; Weiss et al., 2011).
- **Complete assessment:** Reaction time (Reaction Time); Total time (Total Time).

The variables have been transformed with the MinMaxScaler from SciKitLearn library (Pedregosa et al., 2011) to the range between 0 and 1.

### 2.5. Stage 2

#### 2.5.1. Windowing

This windowing differs from the previously performed for segmentation and it was intended to feed the time domain and frequency domain analysis (Section 2.4.1). The size of the window was 64 timestamps with a 50% overlap. The size and overlap were chosen based on the literature recommendations for human activities to capture the temporal dynamics of the signal while ensuring that the data had sufficient resolution for analysis (Banos et al., 2014; Dehghani et al., 2019).

#### 2.5.2. Model inputs

##### 2.5.2.1. Input 1—Time-domain analysis

The **Input 1** of the classificator is the time-domain analysis branch. This branch was feeded with the 64-sample moving window (Section 2.5.1) made with the six sensor signals (three accelerometer axes and three gyroscope axes).

##### 2.5.2.2. Input 2—Frequency-domain analysis

The **Input 2** is the branch for frequency-domain analysis. The input are the windowing signals (Section 2.5.1). We applied the short-time Fourier Transform (STFT) provided by the TensorFlow 2.9.1 framework. All the signals are concatenated as if they were a single signal of 384 samples (6 signals × 64 samples). The STFT is then performed on this new signal with frame length = 20 and frame step = 2 to obtain a spectrogram. Then we applied the logarithm of the magnitude of the Fourier transform.

##### 2.5.2.3. Input 3—Biomechanics variables

The biomechanical variables used were those described in Section 2.4.3.

#### 2.5.3. Classification model

**Figure 4:**
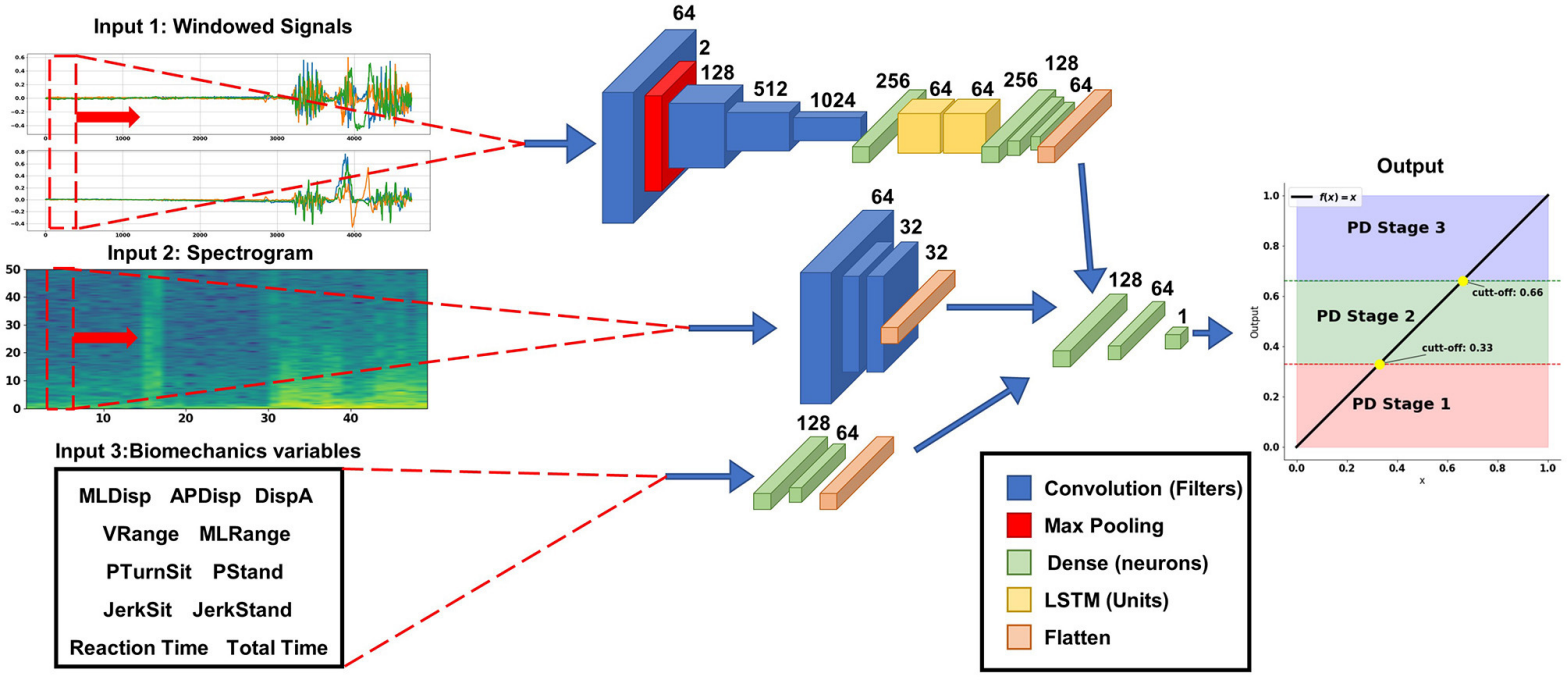
Structure of the Parkinson level classification model with mixed input data. The temporal input data (upper branch) is a moving window of 64 timestamps with the three axes of each sensor (accelerometer and gyroscope); this branch of the model is composed with a series of convolutional layers and LSTM to automatically extract the temporal characteristics of the signals. The branch with the frequency information (center branch) is the spectrogram image of the temporal signal, this branch is composed of convolutional layers to extract the information contained in the images. The branch with biomechanical variables (the lower branch) is composed of densely connected layers. All these branches are joined before the Top Model with a linear output layer between 0 and 1 with the points of 0.33 and 0.66 for the different levels.

Keras API (Chollet et al., 2015) and Tensor Flow (Abadi et al., 2015) 2.0 in Python 3.7.x were used for classification model development (Figure 4).

For **Input 1**, the accelerometer and gyroscope signals were used with a series of 1D convolutional layer concatenations with ReLu activation functions (Rectified Linear Unit), which can extract the features automatically. ReLu is preferred over other activation functions like sigmoid or tanh because it is computationally efficient and avoids the vanishing gradient problem, which can occur when the derivative of the activation function becomes very small (Szandała, 2021). The extracted features were then passed through two Long-Short-Term Memory (LSTM) layers to obtain the signals sequential properties (Matias et al., 2021). Finally, three dense layers with ReLu activation functions were concatenated with the other two input branches.

The **Input 2** the spectrogram image of the signals was used (Ronneberger et al., 2015; Demir et al., 2019), where three 2D convolutional layers with a kernel size of 3 × 3 and ReLu activation functions were concatenated.

For **Input 3** the biomechanical variables were used, and dense layers with ReLu activation function were employed.

Finally, on top of the above networks, two dense layers are used with 128 and 64 neurons with Relu activation function and one output layer with one neuron were used for regression, with a linear activation, to produce a continuous output in the range [0, 1]. The cut-off points for each Parkinson's level were at 0.33 and 0.66.

To compile the model, mean square error was used as the loss measure for the regression problem, and the Adam optimizer.was employed. The evaluation metrics used was “mean square error” which considers the distance between the various categories and imposes a higher error penalty on the categories that are further away from the true value. An iterative design process was performed to fit the model, and the best results were obtained for a configuration with a batch size of 32 for 50 training epochs.

A grid search approach was used to systematically explore different combinations of hyperparameters, such as learning rate, batch size, and number of epochs, and evaluated the model's performance on the training and validation sets. Based on the results of each experiment, the hyperparameters were adjusted, and the process was repeated until the best performance was achieved.

### 2.6. Training, validation, and testing of the classification model

For training and validation the sample has been divided in different dataset:

Firstly, the sample has been divided in two separated datasets. Fifteen participants (five subjects from each group) have been reserved as **test dataset** for testing the classifier. This dataset did not intervened in the training, neither in the validation process. It was just kept apart for the final assessment of performance of the classifier.

The remaining 72 participants composed the training and validation dataset. This dataset was itself divided into three independent folds to perform a stratified three-fold cross-validation (Xia et al., 2020). Two of the three-folds were combined and used in the model training, while the remaining fold was used for model validation. Each training set was resampled and resized using the SMOTE algorithm (Chawla et al., 2002) for the biomechanical variables and with data augmentation (rotating the axes of the sensors artificially 90 and 180°; Pedrero-Sánchez et al., 2022) for the signals, so that the number of instances of each class was approximately balanced. The accuracy and loss evolution plots over the training epochs were obtained.

Once the training was complete, the test dataset was used to evaluate the model performance using a confusion matrix and the geometric mean (G-mean; Kubat and Matwin, 1997).

### 2.7. Sensitivity analysis and comparison with simpler models

To assess the effectiveness of the model topologies identified in the literature and to perform a sensitivity analysis, it is important to evaluate the model's explainability in a clinical setting. Understanding the deep learning model's explainability aids in accurately interpreting the results it generates. To this end, we conducted a sensitivity analysis of the classifier to determine the impact of each input on the model's output.

The sensitivity analysis was performed by making alterations to the inputs and forcing one input to be all zeros when making the inference. This process was repeated for each input. Finally, we compared the outputs obtained for each input variation and analyzed their influence on the output.

Additionally, we used the same training and validation data to train two simplified models based on previous literature: (i) a simplified model that uses only input 1 (which includes convolutional layers and LSTM) called CNN+LSTM (Butt et al., 2020; Xia et al., 2020), and (ii) a simplified model that uses input 1 (including convolutional layers) and input 3 (including dense connected layers) called CNN+biomechanical variables (Pedrero-Sánchez et al., 2022). Input 2 was excluded because no models were found in the literature that used only the spectrogram image as input for Parkinson's disease classification.

We also obtained confusion matrices and mean accuracy for the training and validation folds of these models using the same test dataset.

## 3. Results

### 3.1. Participants

A description of the demographic characteristics and biomechanical variables of the participants, as well as the differences among the HY groups (Table 1).

**Table 1:** Demographic characteristics and biomechanical variables of the participants.

	**All participants**	**HY-I**	**HY-II**	**HY-III**	**ANOVA**
	**(n = 87)**	**(n = 21)**	**(n = 30)**	**(n = 36)**	**(p-value)**
Age (years)	69.09 (8.71)	67.14 (8.20)^*^	66.10 (9.40)^**^	72.58 (7.22)	**0.005**
Weight (*Kg*)	74.41 (15.97)	72.36 (11.88)^***^	85.03 (18.72)^**^	66.75 (9.80)	**< 0.001**
Height (*cm*)	166.14 (8.31)	166.81 (6.92)	170.57 (7.34)^**^	162.06 (7.97)	**< 0.001**
**Sex (** ***n*** **, %)**					
Women	30, 34.48	8, 38.10	5, 16.67	17, 47.22	–
Men	57, 65.52	13, 61.90	25, 93.33	19, 52.78	–
MLDisp (*mm*)	9.29 (7.95)	5.43 (2.65)	8.86 (8.34)	11.89 (8.81)	**0.01**
APDisp (*mm*)	22.90 (11.56)	18.44 (9.52)	21.02 (8.57)	27.07 (13.52)	**0.012**
DispA (*mm*^2^)	773.63 (1191.73)	294.41 (258.85)	717.18 (1251.94)	1100.22 (1379.32)	**0.044**
VRange (*mm*)	24.34 (7.08)	28.34 (6.84)	25.27 (6.58)	21.22 (6.36)	**< 0.001**
MLRange (*mm*)	47.71 (23.75)	49.03 (16.81)	45.12 (24.77)	49.09 (26.60)	0.766
PTurnSit (*W*)	87.41 (42.33)	111.66 (29.56)	96.93 (50.27)	65.33 (29.64)	**< 0.001**
PStand (*W*)	271.03 (86.50)	252.65 (97.75)	236.76 (74.02)	179.81 (76.59)	**0.002**
JerkSit (*m*/*s*^3^)	16.99 (7.40)	16.91 (4.14)	18.34 (7.96)	15.90 (8.35)	0.419
JerkStand (*m*/*s*^3^)	21.66 (11.42)	21.08 (5.90)	24.66 (16.22)	19.51 (8.36)	0.184
TTime (*s*)	14.74 (3.75)	11.83 (1.52)	14.34 (2.66)	16.76 (4.24)	**< 0.001**
RTime (*s*)	1.18 (0.42)	1.03 (0.41)	1.23 (0.49)	1.23 (0.34)	0.147

HY-I, participant in stage according to Hoehn & Yahr; HY-II, participant in stage according to Hoehn & Yahr; HY-III, participant in stage according to Hoehn & Yahr.

MLDisp, range of the Medial-lateral displacement of center of mass (COM); APDisp, range of the Anterior-posterior displacement of COM; DispA, Displacement Area; VRange, range of the Vertical displacement of COM; MLRange, range of the Medial-lateral displacement of COM; PTurnSit, Turn-to-sit power; PStand, Sit-to- stand power; TTime, total time; RTime, reaction time.

Data are expressed as mean (standard deviation).

^*^p < 0.05 between participants with level I and III.

^**^p < 0.05 between participants with level II and III.

^***^p < 0.05 between participants with level I and II.

**Bold** < 0.05 ANOVA between levels I, II, and III.

Table adapted from Mollà-Casanova et al. (2022).

### 3.2. Validation of the segmentation model

**Figure 5:**
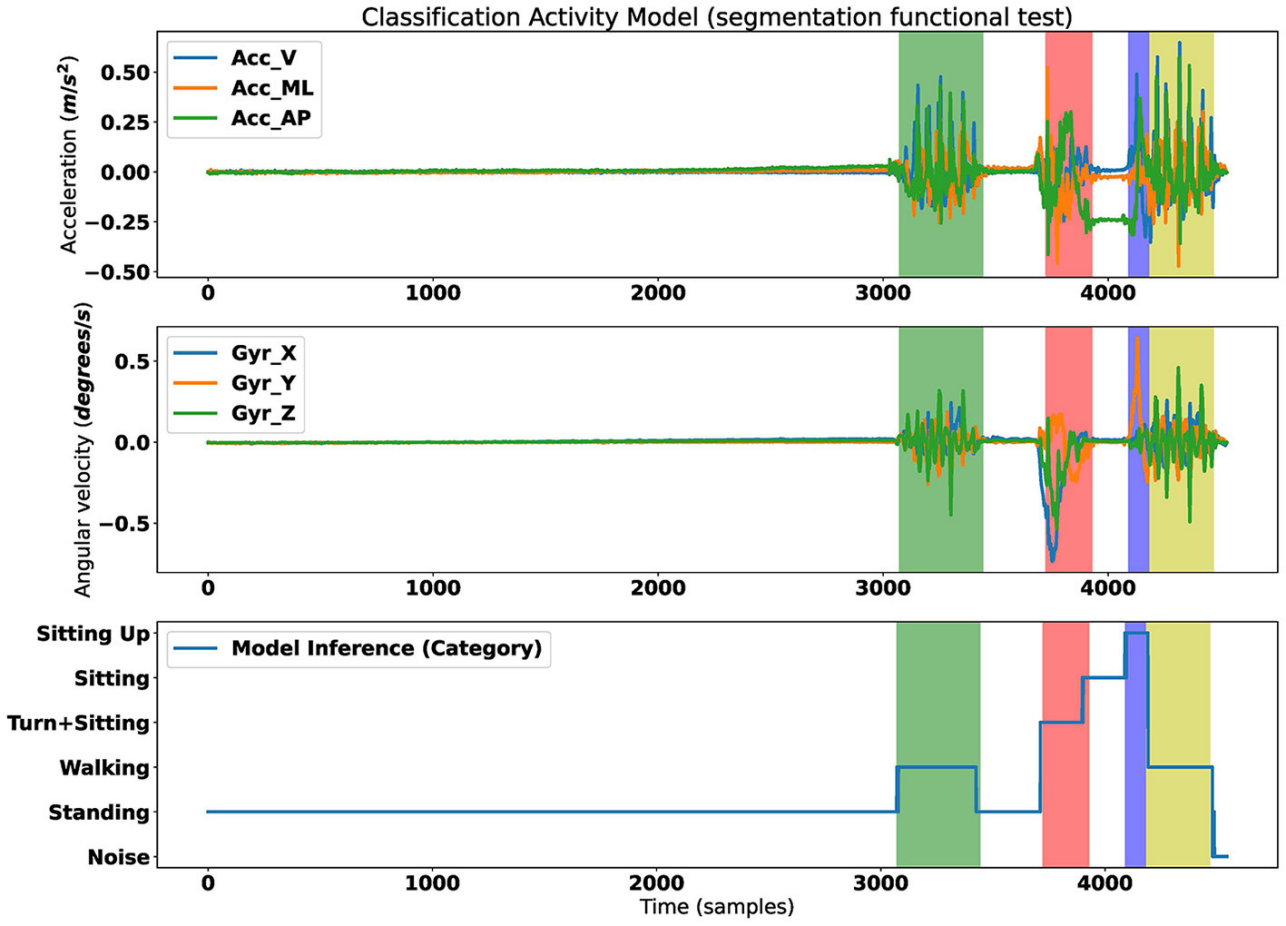
Results of segmentation assessment. **(Top)** Acceleration signal. **(Middle)** Gyroscope signal. **(Bottom)** Result of classification phases of the assessment. Shaded colors are the ground truth segmentation; Green, phase 2 gait; Red, phase 3 turn to sit; Blue, stand from the chair; Yellow, phase 4 gait.

From the second epoch on, the segmentation model achieved an accuracy of 90% and a loss below 0.1. The comparison between the segmentation of the model and a manual segmentation from an expert shows a good agreement (Figure 5).

Therefore, we have used this automatic segmentation to calculate the biomechanical variables and use them as input for the classifier model.

### 3.3. Validation and comparison of the classification models

The accuracy evolution curve during the training of the two-stage classification stabilized at 100% after 5th epoch. The mean of the accuracy results obtained from the three-fold stratified cross-validation for each model in the training and validation phases shown in Table 2.

- CNN + LSTM: 86.42%
- CNN + biomechanical variables: 92.23%
- Proposed Two-stage: 99.64%

**Table 2:** Validation and comparison of the classification models.

	**Three-fold cross (%) validation**	**F1-score (%)**	**G-mean (%)**
CNN+LSTM	86.46	79.00	84.00
CNN+ Biomechanical variables	92.23	81.00	84.00
Proposed two-stage model	99.64	100.00	100.00

**Figure 6:**
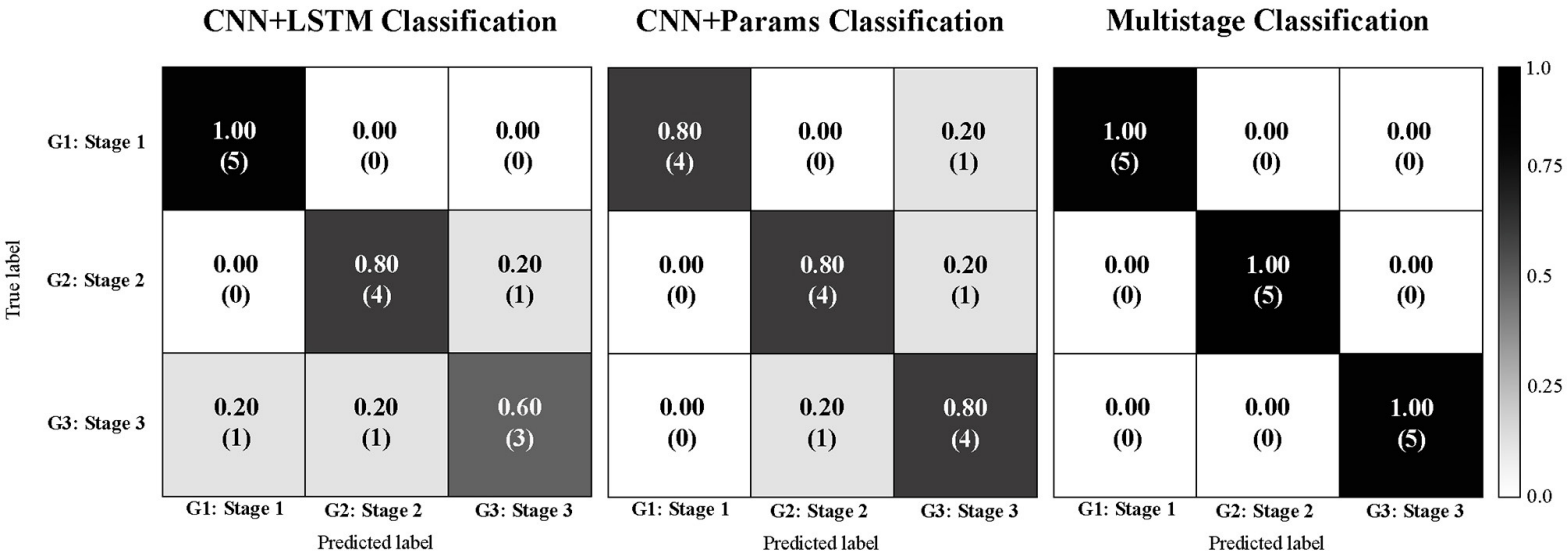
Confusion matrices comparison: Convolutional with Long short-term memory classification Parkinson disease model **(left)**; Convolutional with biomechanical parameters classification Parkinson disease model **(center)**; Proposed two-stage classification Parkinson disease model **(right)**.

The two-stage classification model performed an accurate classification of all the 15 participants of the test sample (Figure 6) and the G-mean obtained was 1.00. Both, the CNN + LSTM and CNN + biomechanical variables achieved a G-mean of 0.84. For, the f1-score, was 0.79 for CNN + LSTM, 0.81 for CNN + Biomechanical variables, and 1.0 for two-stage.

The sensitivity analysis results shows that the major contributions to the model were the image of spectrogram with an accuracy decay of 33.79% (Table 3).

**Table 3:** Sensibility of Stratified three-folds Cross Validation forcing one of the inputs to be all zeros then making the inference with the other two inputs.

	**Mean accuracy**	**Difference with all inputs**
Time-domain analysis	68.97	30.67
Frequency-domain analysis	65.85	33.79
Biomechanics variables	71.23	28.41

**Mean accuracy** is the accuracy in cross validation when the input is set to zero. **Difference with all inputs** is the difference in the mean accuracy obtained with the full input substracting the mean accuracy when the input is set to zero.

For a better understanding of the influence of the anthropometric data in the results, a separate analysis using a standard classifier with only the subject parameters (age, weight, height) as input variables was conducted. The results are presented as Supplementary material.

## 4. Discussion

This paper proposes a two-stage model to classify the early stages of PD (HY-I, HY-II, and HY-III) using a functional assessment test. The test involves the assessment of static balance, gait and lower limb power while sitting and rising from a chair, all within a 2 min timeframe using a single inertial sensor embedded in an Android smartphone (Serra-Añó et al., 2020; Mollà-Casanova et al., 2022).

As already shown in the previous study (Mollà-Casanova et al., 2022), the biomechanical variables obtained from the test are already indicators of disease progression, such as the total time (i.e., Ttime) that increases proportionally. The proposed test provides information on the state of balance MLDisp (*p* < 0.05), APDisp (*p* < 0.05), DispA (*p* < 0.05), gait Vrange (*p* < 0.05), and power in the lower limbs during sit to stand from a chair. There are significant differences (*p* < 0.05) in the biomechanical variables PTurnSit and PStand between the three groups.

The proposed model has been built on two Stages. Regarding Stage 1, the model is able to classify the activity on an instant-by-instant basis, reaching 90% of accuracy from the third epoch onwards. This has been accomplished by utilizing the signals from the inertial sensors and employing semantic segmentation models that have been validated in previous studies for pixel classification in images (Ronneberger et al., 2015) and for electrocardiogram (ECG) analysis (Matias et al., 2021). This semantic segmentation allowed to obtain the signal features that will later be used as input in the classification models. This automatic segmentation has a direct impact on the accuracy of the model. On the other hand, to ensure that all relevant characteristics of the signal in the time domain are captured, one of the input branches of the neural network includes the raw signals themselves, combined with convolutional and LSTM layers of the neural network as Zhao et al. (2018b) and El Maachi et al. (2020), respectively.

With respect to the Stage 2, the proposed model demonstrates a significant improvement in accuracy compared to variables based models in previous studies: 99.64% accuracy using the proposed model, compared to 80% accuracy using SVM, KNN, DT, and RF models (Trabassi et al., 2022). These classifiers have the limitation of using only signal-derived variables, which are clinically relevant for assessing Parkinson's grades, but still have potential for improvement.

When comparing neural network-based classifiers, such as CNN or LSTM, the results are similar, 98% accuracy with CNN (El Maachi et al., 2020) and 92.3% accuracy with LSTM (Butt et al., 2020) and 99% with the combination of CNN and LSTM (Zhao et al., 2018a). Although these results are already very good at classifying PD stages, they have the limitation of only focusing on the time domain. However, it should be noted that in more advanced stages of the disease, certain involuntary tremors may appear, which should be taken into account (Xing et al., 2022). Although some authors have found interesting results analyzing the consequences of tremors using variables in the time domain (e.g., sample entropy; di Biase et al., 2017; Su et al., 2021), the most direct approach would be to consider studying the frequency domain.

Despite the unbalanced training sample, the model responds correctly. To address this issue, training and validation have been carried out using stratified k-fold with artificially augmented data, which allowed balancing and data augmentation to fine-tune the model following the process used in Xia et al. (2020).

Another benefit presented in this paper is the combination of time domain and signal frequency information, along with clinically relevant biomechanical variables selected from the literature. It is worth noting that anthropometric variables of the subjects such as age, sex, height, and weight which have been shown to be important in determining the severity of the diseases (Joshi et al., 2010) have not been used in the classification model. This is because a comparative analysis by group was carried out and there were differences. These variables have been excluded in order to avoid bias in the classification, even though we know that they are important. In this way, the classification model only takes into account the functional test itself (Supplementary material).

The results of our study provide to the scientific community a new model to classify the early stages of PD. The model automatically processes the data recorded by a portable inertial sensor during the execution of a fast an easy functional assessment. Although we do not intend to substitute clinical assessment, we hypothesize that this model may be of interest in the future to better extract functional features in this population, beyond the instability, asymmetry or independence reported in the HY scale. This could lead to more accurate classifications and patient monitoring related to functional capability. To achieve this, further research is needed to validate this new method by comparing it to other clinical scales, such as the PD Questionnaire-8 or the Unified Parkinson's Disease Rating Scale (UPDRS). We believe that detecting different Parkinson's profiles may redefine the stages of Parkinson's and enable anticipation and prevention of its deleterious effects. Additionally, this approach provides a first step toward the development of automated, continuous, and non-invasive monitoring of functionality.

It is important to cautiously interpret the results of this study due to the limitations related to the small sample size. Although the anthropometric parameters were excluded from the model, the differences found between the HY groups could have biased the results. It would be important in future research to consider the use of the modified HY scale, including the intermediate stages (i.e., 0.5, 1.5, and 2.5) to explore the capability of the model to classify all the early-to-moderate stages of the disease. A wider validation including multicentric data, homogeneous samples (regarding anthropometric variables) and additional diagnostic tools would be needed to confirm future clinical applications.

## 5. Conclusion

We show that our two-stage deep learning model can accurately classify people suffering from the first stages of PD. This CNN and LSTM-based technique is more accurate than another parametric technique of machine learning. These results demonstrated that the use of techniques managing raw data, combine with frequency analysis and biomechanical variables, prevents unexpected loss of information. Further, these classification models have been based on the information of a single sensor easily placed on the waist region of the participants in 2 min assessment test. The easy instrumentation required and the short duration of the test make its use feasible in the clinical context.

## Data availability statement

The raw data supporting the conclusions of this article will be made available by the authors, without undue reservation.

## Ethics statement

The studies involving human participants were reviewed and approved by Ethics Committee of Universitat de València (H1517239006520). The patients/participants provided their written informed consent to participate in this study.

## Author contributions

JP-S: conceptualization, methodology, software, and formal analysis. JB-L: resources, conceptualization, supervision, and formal analysis. PS-A: conceptualization, methodology, validation, and investigation. SM-C: investigation and data curation. JL-P: conceptualization, supervision, and project administration. All authors contributed to the article and approved the submitted version.
