# Supplementary material for: Classification of Parkinson's disease stages with a two-stage deep neural network
Source: Front Aging Neurosci. 2023 Jun 2;15:1152917. doi: 10.3389/fnagi.2023.1152917 (PMC10272759; doi:10.3389/fnagi.2023.1152917)
Supplement: Supplementary file 1 [file Data_Sheet_1.PDF]

Supplementary Material 1

1 DISCRIMINANT CLASSIFICATION PARKINSON DISEASE STAGES MODEL WITH ANTHROPOMETRIC DATA

We conducted a separate analysis using a standard classifier (Linear Discriminant Analysis) with only the subject parameters (age, weight, height) as input variables. The same training, validation, and testing process was used as in the proposed model, including Stratified K-Fold cross-validation with 80% of the sample and testing with the remaining 20%. During validation, the model achieved an average hit rate of 0.481.

The classification report for the test results is shown in the table S1:

Table S1. Classification report of the discriminant model of Parkinson's disease stages with the anthropometric data of the participants.

	**Precision**	**Recall**	**F1-score**	**Support**
PD Stage 1	0.00	0.00	0.00	5
PD Stage 2	0.75	0.75	0.75	8
PD Stage 3	0.44	0.80	0.57	5
Accuracy			0.56	18
Macro avg	0.40	0.52	0.44	18
Weighted avg	0.46	0.56	0.49	18

PD: Parkinson disease;
Macro avg: Calculate metrics for each label, and find their unweighted mean. This does not take label imbalance into account.
Weighted avg: Calculate metrics for each label, and find their average weighted by support (the number of thre instances for each label).
Recall: the ability of a classification model to identify all data points in a relevant class;
Precision: the ability of a classfication model to return only the data points in a class;
F1-score: a single metric that combines recall and precision using the harmonic mean.

As can be seen from the results, the model's performance was not particularly good, with an overall hit rate of 0.56 and low precision and recall values for most stages. Therefore, it appears that age, weight, and height alone are not sufficient to differentiate between the different stages of Parkinson's disease.
